# Factors associated with the adoption of a digital health service by patent proprietary medicine vendors (PPMVs) in Lagos, Nigeria

**DOI:** 10.1177/20552076221142666

**Published:** 2022-11-30

**Authors:** Sohail Agha, Laura Alejandra Ruiz-Gaona, Jed Friedman, Nejma Cheikh, Marelize Gorgens

**Affiliations:** 1 Behavior Design Lab, 6429 Stanford University, Stanford, CA, USA; 2 8420 World Bank Group, Washington, DC, USA

**Keywords:** informal providers, PPMVs, digital health, healthcare, LMICs, Nigeria, sub-Saharan Africa

## Abstract

**Background:**

Patent proprietary medicine vendors (PPMVs) are the first point of care for low-income Nigerian households. They are likely to have an important role in a digital care pathway established for low-income Nigerian women and children. Yet, little is known about what drives the adoption of digital platforms by PPMVs.

**Methods:**

This study explores factors associated with the adoption of a digital service, NaijaCare, created to enable PPMVs to increase the range and quality of products and services they offer. A structured, quantitative, face-to-face survey was conducted among 248 PPMVs in Lagos in February and March 2020. Multivariate analysis was conducted to identify factors associated with the adoption of NaijaCare.

**Results:**

Women comprise the majority (67%) of medicine vendors in Lagos. Most medicine vendors (64%) had gotten health training on the job. About a quarter (27%) of medicine vendors reported seeking business advice on the internet. Medicine vendors who had obtained on-the-job training had a 12.31 times higher odds ratio (*p* < 0.01) of adopting the digital service. Medicine vendors who sought business advice on the internet had a 6.48 times higher odds ratio (*p* < 0.001) of adopting NaijaCare.

**Conclusion:**

The study findings suggest that PPMVs’ use of the digital service was driven by their desire to increase business profits. Digital care pathways targeting low-income households should be aligned with the business interests of informal providers.

## Introduction

Since the start of the COVID-19 pandemic, there has been a surge in the introduction of digital health services in Nigeria and growing interest among state governments to incorporate digital tools, including telehealth,^1^ for the provision of primary health care (PHC) services. Before consumers change their health-seeking behavior, however, changes in provider behavior will be needed. For example, the deployment and adoption of digital tools needed for telehealth will require substantial changes in providers’ workflows, including digital referrals, e-prescriptions, the use of diagnostic tools, and electronic medical records. This study describes the profile of PPMVs in Lagos and identifies factors associated with the adoption of a digital service offered prior to the start of the pandemic to increase their business capacity. These findings may help inform the design of telehealth services that integrate informal providers in reaching low-income households in Lagos.

Patent proprietary medicine vendors (PPMVs) are the primary source of medicine for acute conditions in Nigeria. Community-level studies show that PPMVs are the first source of care for up to 55% of childhood illnesses and provide malaria treatment for up to 55% of adults seeking care.^2^ Despite their widespread use by low-income households, PPMVs’ level of health knowledge and treatment practices are often of poor quality.^3^ Improving the quality of services offered by PPMVs and finding an effective way of integrating them into the PHC system remains an important challenge for Nigeria's health system.^3^

Prior interventions to improve the quality of PHC services offered by PPMVs have treated PPMVs as they have treated public health providers: training has been used as the primary means of increasing PPMVs’ capacity to provide better quality health products and services. However, experience suggests that PPMVs do not consider themselves to be healthcare providers *per se*. For example, human-centered design work conducted to develop the NaijaCare digital service showed that PPMVs identify themselves as businesspersons rather than as healthcare providers.^4^ This insight is echoed in the concerns expressed by regulators who feel that PPMVs are primarily interested in business profits and think of themselves as health providers only secondarily.^5^

As shopkeepers, PPMVs sell a broad range of health and non-health products, including medicines, beverages, airtime, and food. Most PPMVs do not have formal training from professional schools of medicine, nursing, or midwifery as is customary for service providers in the Nigerian public sector. PPMVs learn through apprenticeship with other PPMVs^6^ and acquire as-needed on-the-job health training. Their businesses survive on low-profit margins, have little working capital, and are often challenged by cash flow problems. PPMVs have limited capacity to make capital investments needed to improve the range and quality of products and services they offer.

In contrast with traditional classroom training to increase health provider capacity, the NaijaCare digital service allowed PPMVs to take modular, 5-min long, online courses through their existing, internet-enabled, mobile phones. An eCommerce component of the digital service allowed PPMVs to order quality-assured medicine online. This feature enabled medicine vendors to deal with two important limitations of purchasing medicines from the open market: poor quality products and high product prices.^4^ Thus, the digital service enabled PPMVs to have a flexible way of learning how to improve their business skills, increase the quality of products and services they offered, and find solutions to common business-related challenges by getting advice from experienced peers. The aspect of getting peer support is consistent with PPMVs’ socialization into the business.^6^

Interventions that have focused on training informal providers such as PPMVs to improve the quality of services they provide have usually not been designed to address the range of issues confronting a small business. The NaijaCare digital service was designed with the assumption that on-the-job professional education, mentoring, reduced counterfeit drugs through online ordering, and group buying would increase the business capacity and financial viability of informal providers’ businesses and lead to improved quality products and services being offered to low-income households.^4^ With growing interest in the use of telehealth for PHC service delivery in Nigeria, learning from the experience of digital platforms such as NaijaCare - developed to change provider behavior and to increase provide business capacity - is important.

## Methods

### Study design and respondent recruitment

A baseline survey was conducted in 2020 as part of the effort to evaluate the impact of the NaijaCare digital service. Data from this survey allows us to compare early adopters of NaijaCare with geographically co-located PPMVs who had not adopted the service at the time the survey was conducted.

PPMVs were introduced to the digital service during monthly meetings of their professional association, the National Association of Patent Proprietary Medicine Vendors (NAPPMED). The latest news and information relevant to running PPMV businesses are shared during NAPPMED monthly meetings. NAPPMED meetings are held by zones, which may vary substantially from each other, and allow PPMVs with different characteristics to participate. To attend these meetings PPMVs do not have to be registered with the government body that regulates PPMV practice, the Pharmacy Council of Nigeria (PCN). As a result, these meetings are attended by a broad cross-section of medicine vendors in Lagos. Following its introduction in Lagos, about 165 PPMVs self-selected to be part of NaijaCare. Enrollment requirements were having an internet-enabled mobile phone and being older than age 18.

Using stratified random sampling, 58 PPMVs were selected for the survey sample. These PPMVs represented early adopters of the digital service. A listing exercise was conducted using geolocations of adopter outlets: 30 non-overlapping radii of 500 meters each were created containing all 58 PPMVs. The radii length was selected to approximate the size of the PPMV market centered on each selected adopter. A complete listing of PPMVs in these 30 clusters was conducted. A listing of 270 PPMVs located within the 30 clusters was conducted. Following the listing exercise, a structured survey instrument was successfully administered to 248 (92%) PPMVs.

### Survey instrument and ethical clearance

The instrument was developed based on a review of the literature and adapted relevant questions from instruments used in earlier studies conducted with PPMVs in Nigeria. Germane to this analysis, the instrument collected data on socio-demographic characteristics of PPMVs, gender, education, training, registration with the PCN, and membership of NAPPMED. Data were also collected on the sources of advice used by PPMVs.

PPMVs who were owners or managers of their outlet and could be expected to use the digital service were eligible to be interviewed. Participation in the study was voluntary and written informed consent was obtained from all study participants. Ethical clearance was obtained from the National Health Research Ethics Committee (NHREC) of Nigeria in February 2020. The study protocol approval number was NHREC/01/01/2007-12/02/2020.

### Data collection

Data was collected through face-to-face interviews in February and March 2020. The emergence of COVID-19 did not significantly delay the data collection.

### Data analysis

First, since our aim was to identify factors associated with adoption of NaijaCare, a few PPMVs who had registered with NaijaCare but had received training from a separate project were dropped from the analysis (*n* = 11). As a result, the analysis is conducted using data from 237 PPMVs. Second, a comparison of the survey data on PPMVs with data on PPMV registration obtained from the digital service, led to seven PPMVs who were originally classified as being in the comparison group being reclassified as adopters of NaijaCare. This increased the number of adopters of NaijaCare to 65. The remaining 172 PPMVs, who were identified through the listing process, comprise the comparison group. Univariate analysis consisted of description of the characteristics of the sample. Bivariate analysis compared PPMVs who had adopted the NaijaCare digital service with those who had not. Chi-square tests of independence were conducted to determine whether there was any relationship between adoption of the digital service and socio-demographic characteristics, health training, having worked in a PHC center, registration with the PCN, membership of NAPPMED, participation in NGO or government led health trainings, and seeking business advice on the internet.

Multivariate logistic regression analyses were conducted to identify factors associated with the adoption of the digital service. The analyses adjusted for socio-demographic characteristics and other variables. *P*-values were considered statistically significant at *p* < 0.05 for both bivariate and multivariate analyses. The clustering of observations (i.e., the clustering of respondents in sampling units) was taken into account by using the STATA cluster command.^7^

## Results

Column 1 of Table 1 shows the characteristics of the PPMV survey sample. About two-thirds of PPMVs were women. More than half of PPMVs were under 40 years of age. Most PPMVs (70%) had secondary education. About one-fifth of PPMVs had greater than secondary education. The majority of PPMVs (64%) received health training after they had started selling medicines. Nearly half (48%) of PPMVs had ever worked at a PHC center.

**Table 1.** Distribution of sample characteristics and % of PPMVs who adopted NaijaCare.

	(1) Distribution of sample characteristics	Percentage of PPMVs who adopted the digital service		
		(2) % of did not adopt	(3) % who adopted	(4) *p*-value
**Gender**				
Male	32.9% (*n* = 78)	76.9%	23.1%	0.293
Female	67.1% (*n* = 159)	70.4%	29.6%	
**Age**				
Less than 40	56.2% (*n* = 131)	77.9%	22.1%	0.039
40 and older	43.8% (*n* = 102)	65.7%	34.3%	
**Education**				
Primary	8.0% (*n* = 19)	89.5%	10.5%	0.214
Secondary	70.0% (*n* = 166)	71.7%	28.3%	
Greater than secondary education	21.9% (*n* = 52)	69.2%	30.8%	
**Received health training**				
Before started selling medicine	12.2% (*n* = 29)	93.1%	6.9%	<0.001
Never received health training	23.6% (*n* = 56)	91.1%	8.9%	
After started selling medicine	64.1% (*n* = 152)	61.8%	38.2%	
**Ever worked in PHC center**				
No	52.3% (*n* = 124)	69.4%	30.6%	
Yes	47.7% (*n* = 113)	76.1%	23.9%	0.245
**Belong to PCN**				
No	96.6% (*n* = 229)	73.4%	26.6%	
Yes	3.4% (*n* = 8)	50.0%	50.0%	0.145
**Ever received a NAFDAC certification**				
No	73.8% (*n* = 169)	78.1%	21.9%	
Yes	26.2% (*n* = 60)	58.3%	41.7%	0.003
**Belong to NAPPMED**				
No	15.6% (*n* = 37)	100.0%	0.0%	
Yes	84.4% (*n* = 200)	67.5%	32.5%	<0.001
**Staff participated in health training by NGOs in past year**				
No	49.4% (*n* = 116)	82.8%	17.2%	0.001
Yes	50.6% (*n* = 119)	63.0%	37.0%	
**Seek advice on the internet on running a business**				
No	73.4% (*n* = 174)	82.2%	17.8%	<0.001
Yes	26.6% (*n* = 63)	46.0	54.0%	
Total	100.0% (*n* = 237)	72.6%	27.4%	

A small proportion of PPMVs (4%) were members of the PCN, the government body primarily responsible for regulating PPMV practice. A census of PPMVs conducted in 16 states across Northern and Southern Nigeria in 2013–14 found that 12% of PPMVs belonged to the PCN.^8^ About a quarter of PPMVs had received a certification from the National Agency for Food and Drug Administration and Control (NAFDAC), a regulatory body. A recent study found that NAFDAC is able to do more monitoring than other regulatory bodies.^5^ Most PPMVs (84%) were members of the National Association of Patent and Proprietary Medicines (NAPPMED), the professional association of PPMVs. The 2013–14 census found that 81% of PPMVs were part of NAPPMED.^8^ About half of PPMVs reported that staff had ever participated in a health training organized by an NGO or the government. One quarter (27%) of PPMVs reported seeking advice on running a business on the internet.

Columns 2 and 3 of Table 1 show associations between sample characteristics and adoption of the NaijaCare digital service. Overall, 27% of PPMVs adopted the digital service. At the bivariate level, there was no significant difference in the use of the digital service by gender or by the level of education. PPMVs ages 40 and older were more likely to adopt NaijaCare than those under 40 (34% versus 22%, *p* = 0.039). PPMVs who received health training on-the-job were more likely to adopt NaijaCare than those who had been trained before they became PPMVs (38% versus 7%, *p* < 0.001).

Registration with the PCN appeared to be associated with the use of the digital service but the relationship did not reach statistical significance. PPMVs who had received certification from NAFDAC were more likely to use the digital service (42% versus 22%, *p* = 0.003). Membership of NAPPMED was associated with the use of the digital service: 32% of PPMVs who were NAPPMED members used NaijaCare versus 0% of PPMVs who were not (*p* < 0.001). This is probably because NAPPMED meetings provided PPMVs the opportunity to adopt the digital service. However, it is interesting that there was no information spillover to PPMVs who were not members of NAPPMED (i.e., adoption of NaijaCare by non-NAPPMED members).

Participation in a training organized by an NGO or the government was associated with higher use of the digital service (37% versus 17%, *p* = 0.001). There was a powerful bivariate relationship between seeking advice on the internet on how to run a business and use of the digital service: 54% of PPMVs who sought advice on the internet used NaijaCare compared with 18% of PPMVs who did not (*p* < 0.001).

Table 2 shows results from a logistic regression analysis showing the odds of a PPMV using NaijaCare. After adjusting for other factors, female PPMVs became more likely than male PPMVs to adopt the digital service (aOR = 4.14, 95% CI 2.34–7.33). This is primarily because female PPMVs were less likely to seek advice on the internet on running a business (not shown): once getting advice on the internet on how to run a business was added to the model, females became more likely to adopt NaijaCare than males. PPMVs ages 40 and older were more likely to adopt NaijaCare than those younger than 40 (aOR = 2.22, 95% CI 1.01–4.87), even after adjusting for other variables in the model. Secondary education (aOR = 10.68, 95% CI 3.15–36.24) and higher than secondary education (aOR = 9.35, 95% CI 3.20–27.32) were associated with a greater likelihood of adoption of the digital service.

**Table 2.** Logistic regression showing adjusted odds of a PPMV adopting NaijaCare.

	aOR (95% CI)
**Gender**	
Male	1.00
Female	4.14*** (2.34–7.33)
**Age**	
Less than 40	1.00
40 and older	2.22* (1.01–4.87)
**Education**	
Primary	1.00
Secondary	10.68*** (3.15–36.24)
Greater than secondary education	9.35*** (3.20–27.32)
**Received health training**	
Before started selling medicine	1.00
Never received health training	2.02 (0.73–5.61)
After started selling medicine	12.31** (2.45–61.77)
**Ever worked in PHC center**	
No	1.00
Yes	0.43* (0.19–0.97)
**Belong to PCN**	
No	1.00
Yes	2.64 (0.40–17.29)
**Ever received a NAFDAC certification**	
No	1.00
Yes	1.82 (0.98–3.38)
**Staff participated in health training by NGOs in past year**	
No	1.00
Yes	1.15 (0.48–2.71)
**Seek advice on the internet on running a business**	
No	1.00
Yes	6.48*** (3.46–12.16)
Pseudo *R*-squared	29.28%
Number of respondents	225

*p < 0.05, **p < 0.01, ***p < 0.001.

There was a powerful association between PPMVs having received health training after they started selling medicine and their adoption of the digital service (aOR = 12.31, 95% CI 2.45–61.77). A causal inference should not be drawn from this association since reverse causality is possible. NaijaCare adoption was also strongly associated with PPMVs’ seeking advice on the internet on how to run a business (aOR = 6.48, 95% CI 3.46–12.16). By contrast, PPMVs who had worked in a PHC center were less likely to adopt the digital service (aOR = 0.43, 95% CI 0.19–0.97).

Several variables which had a statistically significant relationship with adoption of the digital service at the bivariate level were not associated with the adoption of NaijaCare at the multivariate level. After adjusting for other variables, receiving a NAFDAC certification, and having participated in health training organized by an NGO or the government were no longer associated with the adoption of NaijaCare.

## Discussion

Many low-income households in Nigeria rely on informal providers such as PPMVs for acute care because of absenteeism, drug shortages, and a poor ability to diagnose disease at public sector facilities.^9,10^ Lack of financial access to services due to high user fees at PHC facilities is another barrier to the use of public sector facilities.^3^ Non-poor Nigerian households prefer getting services from private clinics^9^ while poor households choose the convenience, reduced waiting time, availability of drugs, perceived lower cost, and the absence of formality offered by PPMVs.^10^ As their first point of contact for PHC services, individual PPMVs may average 20 or more clients per day compared with a similar average number of clients per day at PHC facilities that have multiple healthcare providers. These factors underline the importance of integrating PPMVs in a digital care pathway established to reach low-income households in Nigeria.

Despite increasing interest in the provision of telehealth services to low-income Nigerian households, little is known about what drives the adoption of digital platforms by PPMVs. This is a subject of some importance given the need to change existing workflows to adapt to a digital care provision pathway. As often-used, culturally accessible, sellers of medicine located in the community, PPMVs could provide their clients the link to better-trained providers at central facilities. Indeed, PPMVs may be interested in offering a link to trained providers as one of the services they offer to the community. While they could provide this link, PPMVs would still need to improve the functions they already perform in the community as drug sellers and providers of advice.

A direct-to-patient^11^ telehealth model is, at present, difficult to imagine as a realistic option for low-income Nigerian households given the low utilization of public health facilities and the absence of trust between households and the PHC system. In addition, the risk of telehealth increasing disparities in healthcare access in LMICs has been highlighted.^12^ A recent study suggests that the initial adopters of teleconsultation services for COVID-19 in Nigeria are high socio-economic status individuals.^1^ A hub and spoke^11^ telehealth model in which a central facility coordinates with a community-based provider such as a PPMV may be able to counteract what might otherwise be a function of a direct-to-patient telehealth model widening disparities in healthcare access.

This study compared PPMVs based in Lagos who adopted a digital service to geographically co-located PPMVs who did not. A minority of PPMVs in the study sample (12%) had obtained health training before they started selling medicine. Nearly two-thirds of PPMVs (64%) obtained health training on the job. There was a large difference (31% points) in the adoption of the digital service between PPMVs who acquired on the job training and those who had acquired health training before becoming PPMVs. There was also a large (36% point) difference in the adoption of the digital service between PPMVs who seek business advice on the internet and other PPMVs. These findings are consistent with what is known about the primary role of PPMVs as shopkeepers who sell medicine and other products for a profit. An observational study of PPMV interactions with clients in Southwestern Nigeria found that selling medicines was the most common (69%) behavior that PPMVs engaged in, while giving advice to customers (30%) and asking questions about their illness history (19%) were less common.^13^ These findings are also consistent with research conducted at the design phase of the digital service, which showed that PPMVs consider themselves primarily as businesspersons.^4^

That female PPMVs became more likely than male PPMVs to adopt the digital service once the behavior of seeking advice on the internet was adjusted for in the multivariate model, suggests that gender-related, normative, barriers to provider adoption of digital platforms may play an important role. The finding that older providers were more likely to adopt the digital service is interesting. It is often assumed that younger, more tech-savvy individuals, are the first to adopt digital services. That older PPMVs were more likely to adopt NaijaCare may reflect a greater recognition on their part of the importance of taking advantage of offers that may help build their business. The association between secondary or high education and adoption of NaijaCare was powerful, suggesting that PPMVs with less than secondary education may require additional support in adopting digital platforms.

The strengths and limitations of this study should be kept in mind when interpreting its findings. An important limitation of this study is the cross-sectional nature of the data used for the analysis. As a result, no causal inferences can be drawn regarding the direction of the relationship between variables. A strength of the study is that it provides a representative sample of PPMVs in 30 local markets in Lagos, each with a radius of 500 meters, representing a substantial area within Lagos.

When mHealth has been used to increase the capacity of public sector providers in Nigeria, it has usually relied on a single strategy such as training.^14,15^ mHealth training efforts in Nigeria have focused on midwives or community health extension workers (CHEWs), who obtain 2–3 years of health training before providing health services. We are not aware of prior efforts to use mHealth to increase the business capacity of informal providers in Nigeria. In comparison with trained public sector service providers, informal providers such as PPMVs face a range of barriers to the provision of quality products and services that go well beyond training. For this reasons, multiple digital strategies implemented simultaneously, including training, online ordering, group purchasing, and access to working capital – which solve for the range of business problems faced by small businesses – are likely to make it more attractive for PPMVs to adopt a digital service. Solving for critical business problems faced by informal providers is likely to have an impact on the quality of products and services offered by them. Increased business capacity and greater business stability are more likely to lead to improvements in the range and quality of health products and services offered by PPMVs to low-income clients.

The role of PPMVs’ professional association, NAPPMED, should be recognized. Regulators from the primary agency responsible for regulating PPMV practice, PCN, and NAFDAC report not trusting PPMVs because they are too “business-minded” and because they see themselves only secondarily as healthcare providers. By contrast, NAPPMED provides business and social support to PPMVs. It monitors members’ activities, including stocking and sale of medicines, discourages sale of counterfeit drugs, and provides cash and in-kind assistance for members in times of hardship. One study found that 90% of PPMVs were part of NAPPMED and 86% actively participated in NAPPMED-supported activities.^5^ Peer mentoring activities of NAPPMED are accepted by PPMVs, while a tense relationship have been noted between PCN and PPMVs.^10^ A mechanism for formally recognizing NAPPMED-supported activities and strengthening NAPPMED's capacity to monitor quality of products and services offered by PPMVs may increase PPMVs’ adherence to quality standards and strengthen PPMV businesses.^8^ This could be especially important given that regulatory agencies do not currently have sufficient human or financial resources to monitor PPMV practice and tend to approach PPMVs with a high level of distrust.^5^

The negative association between having worked at a PHC center and adoption of the digital service is consistent with our conclusion: PPMVs’ interest in their business and professional growth are more likely to drive digital platform adoption than their interest in becoming better providers of health services *per se*. This finding may also reflect the fact that PPMVs who have worked in the public sector may have lower levels of digital literacy than other PPMVs.^16^ Overall, the study findings support the conclusion that efforts to integrate PPMVs into a digital care pathway that provides PHC services to low-income households in Lagos should appeal to the business and professional development interests of PPMVs.

This is one of the first studies to provide insights into the adoption of a digital health platform by informal providers in Nigeria.^17^ Early adopters of technology are often those who have greater social and economic status and can risk failure because their financial status can help them absorb the risk.^18^ A more complete understanding of early and later stages of digital technology adoption by informal providers in Nigeria is needed to better contextualize the findings of this study. Finally, our findings are specific to PPMVs in Lagos and should not be generalized to other parts of Nigeria. Studies have shown that the proportion of PPMVs with heath training is much greater in the North than in the South: a recent study found that 38% of PPMVs in the North had medical training compared with 11% of PPMVs in Lagos.^10^ It is possible that factors that drive providers in Northern Nigeria who have obtained 2–3 years of health training before they become PPMVs are different from what drives PPMVs in Lagos to adopt a digital platform.
